# Behavioural insights in the underuse of cardiac resynchronisation therapy in heart failure: a pilot survey on incentive perceptions among referring cardiologists

**DOI:** 10.1186/s13561-025-00657-0

**Published:** 2025-07-18

**Authors:** Joan Costa-i-Font, Georgiana Miler-Raicu, Elena Arbelo, Ruben Casado-Arroyo, Aya Sami, Eric Wei Seong Tee, Joseph Hazel, Laurent Roten, Tobias Reichlin, Haran Burri, Khaled Albouaini, Nikola Kozhuharov

**Affiliations:** 1 London School of Economics and Political Science, London, UK; 2 Department of Cardiology, Inselspital, University Hospital Bern, Freiburgstrasse 20, Bern, 3010 Switzerland; 3 Arrhythmia Section, Cardiology Department, Hospital Clínic, University of Barcelona, Barcelona, Spain; 4 Institut d’Investigació August Pi i Sunyer (IDIBAPS), Barcelona, Spain; 5 Centro de Investigación Biomédica en Red de Enfermedades Cardiovasculares (CIBERCV), Madrid, Spain; 6 European Reference Network for Rare, Low Prevalence and Complex Diseases of the Heart - ERN GUARD-Heart, Amsterdam, The Netherlands; 7 Department of Cardiology, H.U.B.-Hôpital Erasme, Université Libre de Bruxelles, Rte de Lennik 808, Bruxelles, 1070 Belgium; 8 Cardiac Pacing Unit, Cardiology Department, University Hospital of Geneva, Geneva, Switzerland; 9 Royal Liverpool University Hospital, Liverpool, UK; 10 Liverpool Heart and Chest Hospital NHS Foundation Trust, Liverpool, UK; 11 Department of Cardiology, Cardiovascular Research Institute Basel (CRIB), University Hospital Basel, University of Basel, Basel, Switzerland

**Keywords:** Behavioural health economics, Choice architecture, Cognitive biases, Social incentives, Heart failure, Cardiac resynchronisation therapy

## Abstract

**Aims:**

Heart failure is a leading cause of hospitalisation in patients over 50, significantly impacting both quality of life and survival. Despite the well-established benefits of Cardiac Resynchronisation Therapy (CRT), its utilisation in clinical practice remains suboptimal. Traditional incentives, have shown limited effectiveness in increasing CRT referrals. This manuscript explores how behavioural economics can offer a novel framework for improving CRT uptake by leveraging behavioural incentives, particularly choice architecture and social incentives, to influence physician referral patterns.

**Methods and results:**

We underscore key concepts of behavioural economics, including choice architecture (nudges, reference points, sludges), cognitive biases (status quo bias, overconfidence bias, availability bias), and social incentives, which are applied in designing incentives to promote CRT referrals. A survey was conducted with 51 physicians from six European countries, including electrophysiologists, heart failure specialists, and general cardiologists, recruited through cardiology networks and personal contacts. Participants rated their perceptions of five incentive strategies using a Likert scale (1–5). Behavioural incentives, such as peer comparison through league tables (social incentive) and decision prompts in electronic health records (choice architecture nudge), were perceived as more effective than traditional financial incentives, with a median Likert score of 4.0 [IQR 3.0–5.0] versus 2.5 [IQR 1.5–3.0] for traditional incentives (*p* < 0.001).

**Conclusions:**

These findings suggest that interventions drawing on behavioural economics, particularly those utilising social incentives and choice architecture redesign, may offer more effective to increasing CRT referrals than traditional incentives. Such interventions could enhance CRT uptake and outcomes for heart failure patients.

**Graphical Abstract:**

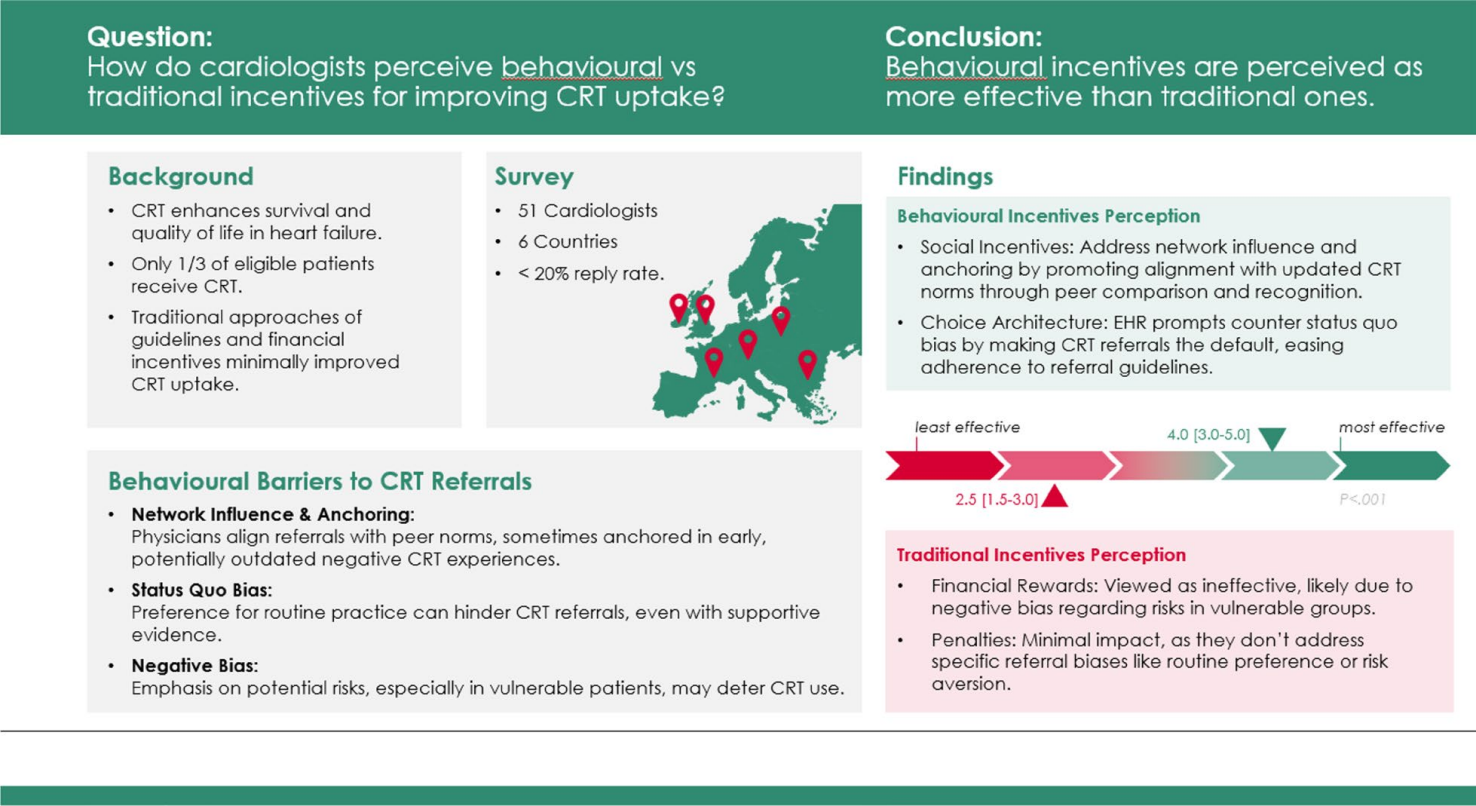

**Supplementary Information:**

The online version contains supplementary material available at 10.1186/s13561-025-00657-0.

## Lay Summary

Heart failure is a leading cause of hospital admissions for people over 50 and significantly impacts patients’ quality of life and life expectancy. Cardiac Resynchronization Therapy (CRT) is a treatment that helps the heart pump more effectively in people with certain types of heart failure. Despite its apparent benefits, many patients who could benefit from CRT do not receive it. This study explored why this happens and new ways to encourage doctors to refer more patients for CRT. This survey asked doctors to share their opinions on ways to increase CRT referrals. These methods included traditional incentives like financial rewards and penalties and behavioural approaches like peer recognition and nudges. The results showed doctors preferred behavioural incentives, such as reminders and professional recognition, over financial rewards or penalties. These findings suggest doctors are more likely to increase CRT referrals when motivated by social incentives and nudges rather than financial rewards. This information will be used to design better strategies to ensure more patients receive the needed treatment. Future research will focus on testing these strategies to improve CRT referrals and ultimately help more heart failure patients.

## Key Learning Points

**What is already known:**

Cardiac Resynchronization Therapy (CRT) is an effective treatment in certain heart failure patients. It improves the quality of life and survival rates. Despite clear guidelines and evidence supporting CRT, its uptake remains suboptimal, with only one-third of eligible patients receiving the treatment. Traditional strategies to increase CRT referrals, such as financial incentives and clinical guidelines, have had limited success in improving referral rates.

**What this study adds:**

Physicians rated behavioural incentives, such as social recognition and choice architecture nudges, as more effective than traditional financial incentives or informational campaigns in increasing CRT referrals. This study suggests that addressing cognitive biases and employing behavioural strategies may significantly improve CRT utilisation. These insights lay the groundwork for developing and testing new incentive-based interventions to boost CRT uptake and enhance patient outcomes.

## Introduction

### CRT and past efforts to improve uptake

Heart failure is a leading cause of hospitalisation in patients over 50, significantly impacting both quality of life and survival. Cardiac Resynchronization Therapy (CRT) is an established treatment for patients with heart failure and concomitant electrical conduction abnormalities [1]. CRT is typically indicated for patients with reduced left ventricular ejection fraction (< 35%), left bundle branch block, and symptomatic heart failure despite optimal medical therapy [2, 3]. Observational data suggest that approximately 5–10% of all heart failure patients ultimately meet the full eligibility criteria for CRT. This corresponds to an estimated 400 potential CRT candidates per million inhabitants annually in European countries [4–6].

**CRT** functions by synchronising left ventricular contractions **through biventricular or conduction system pacing** (CSP), correcting left bundle branch block-induced dyssynchrony [7–9]. This treatment is intended to improve left ventricular function and enhance overall haemodynamic status [2, 3].

CRT device implantation is generally performed as a day-case procedure under local anaesthesia with possible mild sedation. The procedure has a well-established safety profile and is regarded as highly cost-effective.^2^ Beyond its capacity to improve cardiac function, CRT has demonstrated substantial benefits in reducing morbidity hospitalisation rates and improving survival and quality of life in eligible heart failure patients [2, 3]. CRT is delivered through either CRT pacemakers, which provide resynchronisation alone, or CRT defibrillators, which also offer protection against sudden cardiac death through defibrillation. While CRT defibrillators are associated with higher upfront costs, they are considered cost-effective within their indicated populations. The initial clinical indication typically determines CRT eligibility, with the decision between CRT pacemaker and defibrillator guided by individual risk stratification for sudden cardiac death [4].

Despite robust evidence supporting its use and clear guideline recommendations, CRT remains underutilised in clinical practice, with only one-third of eligible patients receiving the therapy across Europe [4, 10–13]. This underuse suggests that barriers beyond clinical eligibility, such as behavioural factors, play a significant role in limiting CRT uptake.

Previous efforts to improve CRT uptake have primarily focused on professional societies’ guidelines dissemination and recommendations for CRT implantation [2, 3, 12, 14]. Healthcare systems and insurers align reimbursement policies with these guidelines, effectively creating financial incentives for CRT implantation. However, CRT referral rates remain suboptimal despite these incentives, suggesting that professional societies’ guidelines and financial incentives alone are insufficient to drive uptake [10, 12, 15–18]. Guideline dissemination aims to increase cardiologists’ awareness of CRT’s indications, benefits, and procedural details. However, research shows that knowledge resistance and behavioural biases can hinder physicians from adopting evidence-based practices [16–21], highlighting the need for more comprehensive approaches. This challenge is also recognised in a joint position paper by the Heart Failure Association, the European Heart Rhythm Association, and the European Association of Cardiovascular Imaging, which calls for action to address CRT underuse through four domains: (1) increasing referrals, (2) improving candidate selection, (3) reframing “non-response” as disease modification, and (4) enhancing post-implant care. Our study aligns with this framework by targeting referral behaviour through behavioural economic insights [4].

Additionally, the **growing clinical interest in CSP** presents a timely opportunity to revisit and improve CRT uptake. While CSP is not a replacement for biventricular pacing in CRT, the momentum surrounding its adoption **can serve as a catalyst to address some of the long-standing behavioural barriers** that have historically limited referrals for CRT [7–9]. However, CSP itself also introduces new challenges for CRT uptake that warrant timely consideration. CSP implantation time in heart failure patients may be comparable to conventional CRT procedures, and reimbursement is often limited, frequently aligned with that of standard pacemakers, despite the increased technical complexity. This underscores the importance of timely acknowledging incentive misalignments in reimbursement while leveraging the current momentum to implement behavioural strategies that promote CRT adoption.

### Current practice for referrals and challenges

Even though the CRT indication is well-defined, and the required information is readily available during patient consultation [2, 3], cardiologists are unsure when and how to refer patients for CRT [10]. This phenomenon may result in hesitation in recommending this treatment option. Despite strong scientific evidence, general cardiologists are often sceptical that CRT can significantly enhance outcomes. This phenomenon may be due to knowledge resistance [19, 22].

**Knowledge resistance** is a psychological phenomenon where individuals may resist accepting new information or modifying their beliefs even in the presence of robust evidence [22]. It has an evolutionary explanation to strengthen group bonds and thereby create “knowledge tribes” with distinct beliefs among those using alternative clinical practices. Knowledge resistance may manifest as a reluctance to adopt specific practices or learn new evidence, even when physicians have access to the necessary evidence [19, 22]. This challenge requires a deeper understanding of the cognitive biases and behavioural barriers underlying CRT referral decision-making.

**Bias in learning**,** specifically negative bias**, can also prevent physicians from referring patients for CRT [19]. Negative bias refers to the tendency to focus more on potential harms or adverse outcomes rather than benefits [16, 17]. Similarly, healthcare professionals often prioritise safety concerns and explicitly react to more immediate or prominent dangers. Such biases can affect referrals for CRT, as physicians may overemphasise some risks associated with the procedure, such as complications or ineffectiveness, and undervalue its potential benefits, thereby increasing the risk perception and delaying or preventing appropriate referrals.

### Traditional incentives vs. behavioural incentives

To further address the underuse of CRT, cardiology societies and health systems have relied more widely on traditional incentives, such as financial incentives associated with clinical activity for CRT evaluation, implantation, and follow-up, as well as information provisions in the forms of guidelines and recommendations for referring physicians [2, 3, 12, 14]. In this context, financial incentives typically refer to reimbursement linked to outpatient activity rather than direct referral payments. In Switzerland, for instance, CRT-related evaluation often includes multiple GP outpatient visits for guideline-directed medical therapy up-titration, along with baseline and follow-up transthoracic echocardiography within 2–3 months. A single echocardiogram is reimbursed at approximately 400 EUR [23]. Although referral rates in the UK’s NHS are comparable, in contrast, financial incentives in NHS are more limited due to GP global budgeting, which may constrain outpatient activity and referral behaviour. Consistent with this, behavioural economics research suggests that relying solely on traditional incentives may not give rise to the desired outcomes [16–19]. The latter is especially true when individuals do not manage to learn the latest information available as it requires effort or are subject to an array of cognitive biases. Furthermore, individuals’ learning is not independent of the group’s behaviour.

Behavioural learning theories provide valuable insights into incentivising treatment uptake by taking advantage of cognitive biases influencing individuals’ decision-making processes, e.g., making information more accessible to digest, salient, or adopted by other patients. These theories emphasise that heuristics, social influences, and emotions determine human behaviour [18, 19, 24]. Often, incentives work solely as signals of what others do or what is desirable for individuals to do. Hence, key behavioural learning concepts which must be considered to enhance CRT uptake include **cognitive biases.**

In the context of CRT referrals, among several learning biases, including a negative bias, **overconfidence** in one’s clinical judgment may lead to under-referral due to unwarranted certainty in the course of disease progression or expected patient outcomes. **Anchoring bias**, or the tendency to rely heavily on some initial information, might also contribute to under-referral if a physician’s early experiences with CRT were negative or less effective than expected [19, 24]. This bias can be compounded if physicians have missed recent technical advances in biventricular pacing, the option for conduction system pacing [7, 8], and improvements due to operators’ accumulated experience and skills. Consequently, outdated perceptions may persist, limiting referrals despite advancements that have enhanced CRT’s efficacy and safety. Consequently, interventions must address these cognitive biases and influence physicians’ decision-making to enhance CRT referrals.

**Traditional incentives**, such as financial rewards for referring CRT patients, may not make a significant difference. Furthermore, **incentives backfire** when physicians are reluctant to change their referral patterns [19, 25, 26], even in the presence of financial incentives, when incentives might not compensate for the (administrative and cognitive) costs of behavioural changes, the so-called **sludge**. This might be due to hassle cost and factors such as time constraints, comfort with familiar practices, or perceived complexities associated with CRT referrals [19].

**Incentive backfiring** occurs when an incentive to drive a particular behaviour unintentionally produces the opposite effect. For instance, blunt monetary rewards for CRT referrals may inadvertently reduce intrinsic drive when the task of patient selection is otherwise perceived as interesting and could undermine moral considerations [19, 25, 26].

In contrast with traditional monetary rewards, behavioural incentives can encourage desired behaviours by addressing cognitive biases and social motivations. For instance, sometimes, a small financial incentive can be effective when used as a **commitment device together as a secondary incentive** within a behavioural incentive design [16, 17, 19]. One example of leveraging **loss aversion** in healthcare is the prefunded pay-for-performance model, where physicians receive an upfront portion of their expected incentive payments but must return unearned amounts if they do not meet predetermined performance targets. This approach, tested in a physician network, aimed to enhance adherence to evidence-based practices by capitalising on the psychological principle that losses weigh more heavily than equivalent gains [27]. While such models have not been used for CRT referrals, they illustrate how financial commitment mechanisms might help mitigate behavioural barriers to guideline adherence.

Besides adjusting incentives for cognitive biases such as loss aversion, one needs to consider that practitioners are sensitive to their social image and the same in failing to fulfil the commitment and then must return the pre-payment.

Healthcare professionals suffer further bounded learning due to time and attention constraints, limiting their ability to fully grasp and effectively implement new information like studies and guidelines. When considering CRT, these professionals may demonstrate the **availability heuristic (supply trigger)**, leading to recall experiences that were negative or less effective than expected [22, 28]. Consequently, they become trapped in the default heuristic, displaying **status quo bias** and refraining from referring CRT, as they perceive it potentially harming patients. **Rise aversion**, a behavioural characteristic of health care professionals, also influences their decision-making, with a tendency to recommend treatments that offer a higher likelihood of patient survival [22]. They may avoid referring CRT to protect patients from potential risks. These biases can lead to systematic errors in belief and decision-making.

### Social incentives and motivation in CRT referrals

Social incentives refer to signals influencing an individual’s desire for esteem, self-understanding, and positive self-image [19]. Social incentives are crucial in shaping individual behaviours within groups, including CRT referrals. For instance, physicians may be more likely to refer patients for CRT if they perceive positive social recognition from their colleagues or the medical community. Officially endorsed league tables indicating the number of patients referred by cardiologists could be used as a social incentive. Social incentives lead to **social multipliers**, indicating how many other physicians may increase their referrals for CRT procedures by following the example of a single physician in their peer network. For instance, **socialisation biases** may also lead to conforming with prevailing referral practices within the cardiologists’ professional circles, whether positive or negative. To enhance this effect, regular meetings for continuous medical education and discussions about CRT referrals among cardiologists in the region could be beneficial [19].

Social incentives result from social constraints to behaviour, including the presence of social norms, which are transformed through new **narratives**. It is challenging to change the behaviour of the large group of cardiologists who do not refer CRT candidates due to the disbelief in the effectiveness of the treatment. A narrative can be created to highlight the evidence supporting CRT to influence this norm. An example of a narrative to reduce over-prescription of antibiotics is “Not All Bugs Need Drugs” [29]. In the case of cardiac resynchronisation therapy, a narrative could use a directive and memorable slogan encouraging CRT uptake. The narrative would be created by role models within the group, using a process known as norm persuasion. For instance, “CRT: Consider, Refer, Treat” reinforces the importance of timely referrals for eligible patients. Promoting this narrative in conferences, guidelines, and clinical discussions can strengthen the perception of CRT as a proactive treatment strategy. As more individuals adopt this new norm, it will begin to spread and ultimately lead to a change in the CRT referral practice [19].

Similarly, a way to change social norms is by taking advantage of **tipping points**, namely the critical mass of behavioural change needed to trigger a broader and lasting shift in practice. By identifying and strategically leveraging these tipping points, interventions can be designed to create a cascading effect in CRT referrals, ultimately leading to a significant increase in uptake [19].

### Choice architecture: nudges and sludges in CRT uptake

The concept of choice architecture originates from behavioural economics and refers to how decisions are influenced by how choices are presented. Its primary objective is to enhance decision-making and to drive desirable behaviour without removing any options or significantly changing economic incentives [19, 30]. Within healthcare and in the context of CRT, choice architecture can be strategically utilised to influence clinician and patient behaviour.

Our perceptions and decisions are often influenced by **reference points**, which serve as mental benchmarks or comparison standards [19, 30]. For example, a target number of CRT referrals could serve as a reference point for physicians. Studies have shown that deviations from these reference points are perceived as losses, enhancing motivation due to loss aversion.

The **status quo** effect and inertia highlight our natural tendency to maintain current behaviours or choices [31]. This can be effectively targeted by interventions such as nudges and defaults. **Nudges** are subtle changes in how options (the choice architecture) are presented, aiming to guide individuals towards a desired choice without removing any possibilities [19, 30]. A good example is the setting of CRT referral as the default option in electronic health record systems, which aims to capitalise on the status quo bias. Alternatively, **sludges** are barriers to certain behaviours, thereby discouraging them [19]. In the case of CRT, increasing the complexity of opting out of referral could act as a sludge. For example, requiring cardiologists to justify non-referral in electronic health records could introduce friction and encourage guideline adherence.

A potential strategy to mitigate learning biases and heuristic-driven behavioural barriers among healthcare professionals regarding CRT referrals is to implement Continuing Medical Education events. These top-down initiatives, such as lunch meetings or workshops, can feature expert-led lectures from cardiologists who share evidence-based guidance in their own experience and provide the latest updates on the benefits and safety of CRT. Furthermore, such events may positively influence clinical decision-making through the **bandwagon** effect, encouraging healthcare professionals to adopt best practices in CRT referrals, especially when they see widespread acceptance of these practices among their peers [19].

One way to use social influence on nudges is by using **meta-nudges**, which involve social norms or the influence of respected figures to drive behaviour [19, 32]. For instance, endorsements from leaders in the cardiology community can significantly influence referral behaviour, emphasising the impact of societal influence on individual choices. In addition to nudges and sludge, other interventions like decision aids designed to outline CRT benefits and risks can aid in informed decision-making. Streamlining the referral process, **enhancing ease and convenience**, can further remove barriers and increase CRT referrals [19].

## Methods

### Recruitment for a pilot survey on behavioural interventions

A pilot survey targeting CRT-referring physicians across Europe was conducted to assess the perception of behavioural interventions on CRT referrals. Participants were recruited via cardiology department mailing lists and professional personal contacts. Email invitations containing a link to the online survey hosted on Google Forms were sent to over 400 contacts. However, the exact number of recipients is uncertain, as initial participants were encouraged to share the survey with colleagues involved in managing patients with CRT indications. No follow-up reminders were issued to encourage survey completion.

### Survey questionnaire

This simple 2-minute survey comprised five questions, each evaluating the effectiveness of specific interventions designed to increase CRT referrals. Clinicians’ perceptions of the five different behavioural and traditional incentive strategies were assessed using a Likert scale from 1 to 5, where 1 indicated the lowest perceived impact and 5 the highest possible positive impact on CRT uptake. Additionally, the survey provided a free-text option for participants to suggest the most effective strategies for improving CRT referrals.

Two questions addressed traditional incentives, monetary rewards and penalties (Questions 1 and 4), while the remaining three focused on behavioural incentives, such as choice architecture with nudges (Question 2) and social incentives (Questions 3 and 5). After the first 10 participants completed the survey, a question about the participants’ country of practice was added to facilitate analysis of potential regional disparities in responses.

### Ethical approval

The survey was fully anonymised, with no identifiable personal or patient data collected. Thus, ethical approval was not required. The study adheres to the principles outlined in the Declaration of Helsinki.

### Data analysis

Each respondent was anonymised and assigned a unique identifier. The questionnaire results were summarised using frequency distributions of Likert scale responses, displayed as histograms. Visual inspection of the histograms was used to assess the normality of data distribution. Medians and interquartile ranges were calculated to summarise the central tendencies of the responses. For comparison, responses related to traditional and behavioural incentives were aggregated. The Wilcoxon signed-rank test was employed to analyse the differences in medians between questions on behavioural and traditional incentives. All statistical analyses were performed using SPSS version 28.0 (IBM Corporation, Armonk, NY, USA).

Results.

**Fig. 1:**
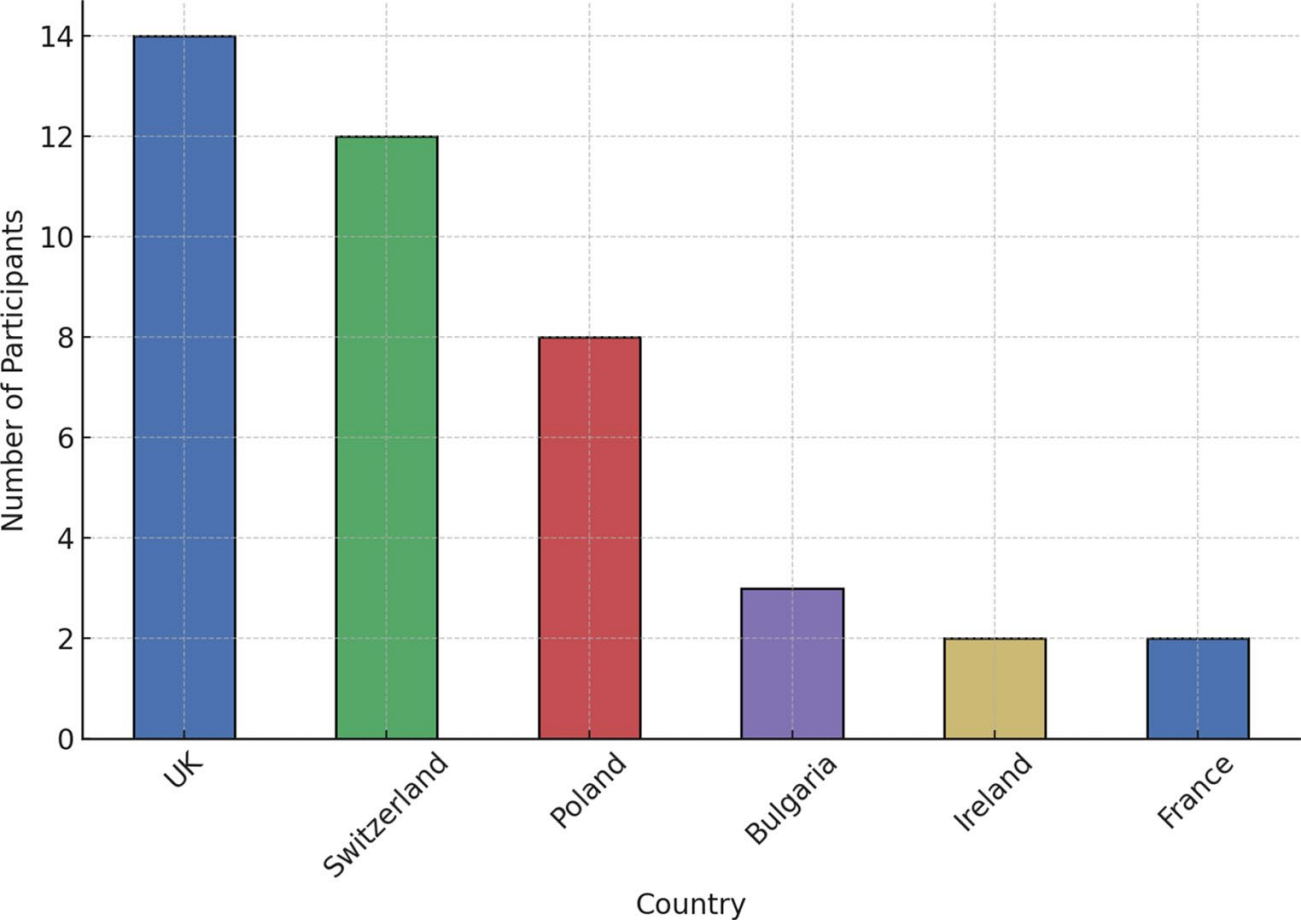
Country of practice of the participants*. *This question was added to the survey after responses from the first 10 participants had already been collected

Fifty-one physicians from 6 European countries (Fig. 1) participated in the survey. Participants included a mix of electrophysiologists, heart failure specialists, and general cardiologists. Invitations were sent to over 400 physicians. Accordingly, the response rate was lower than 20%. The most significant number of responses came from the UK (34.1%), Switzerland (29.3%), and Poland (19.5%).

**Fig. 2:**
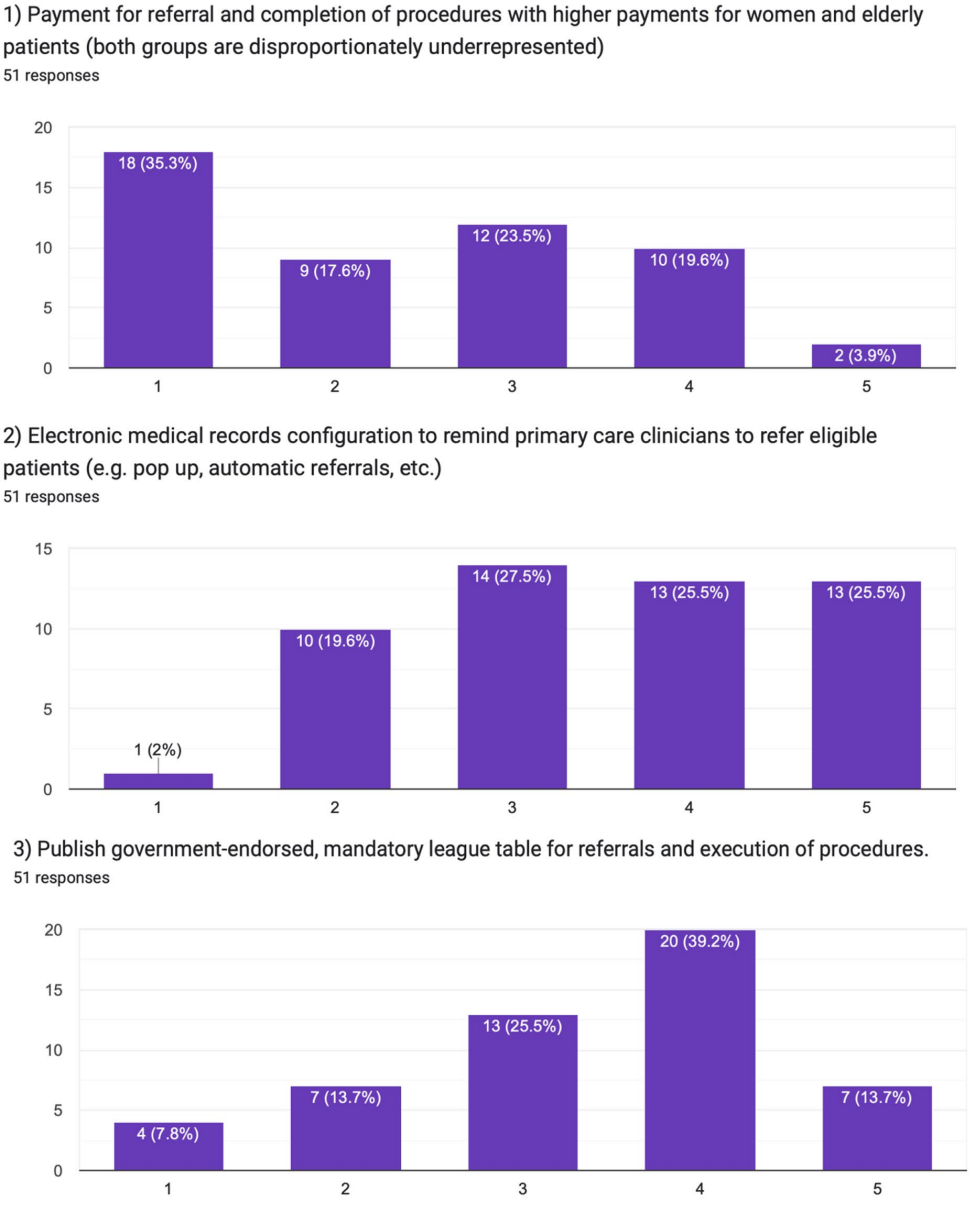

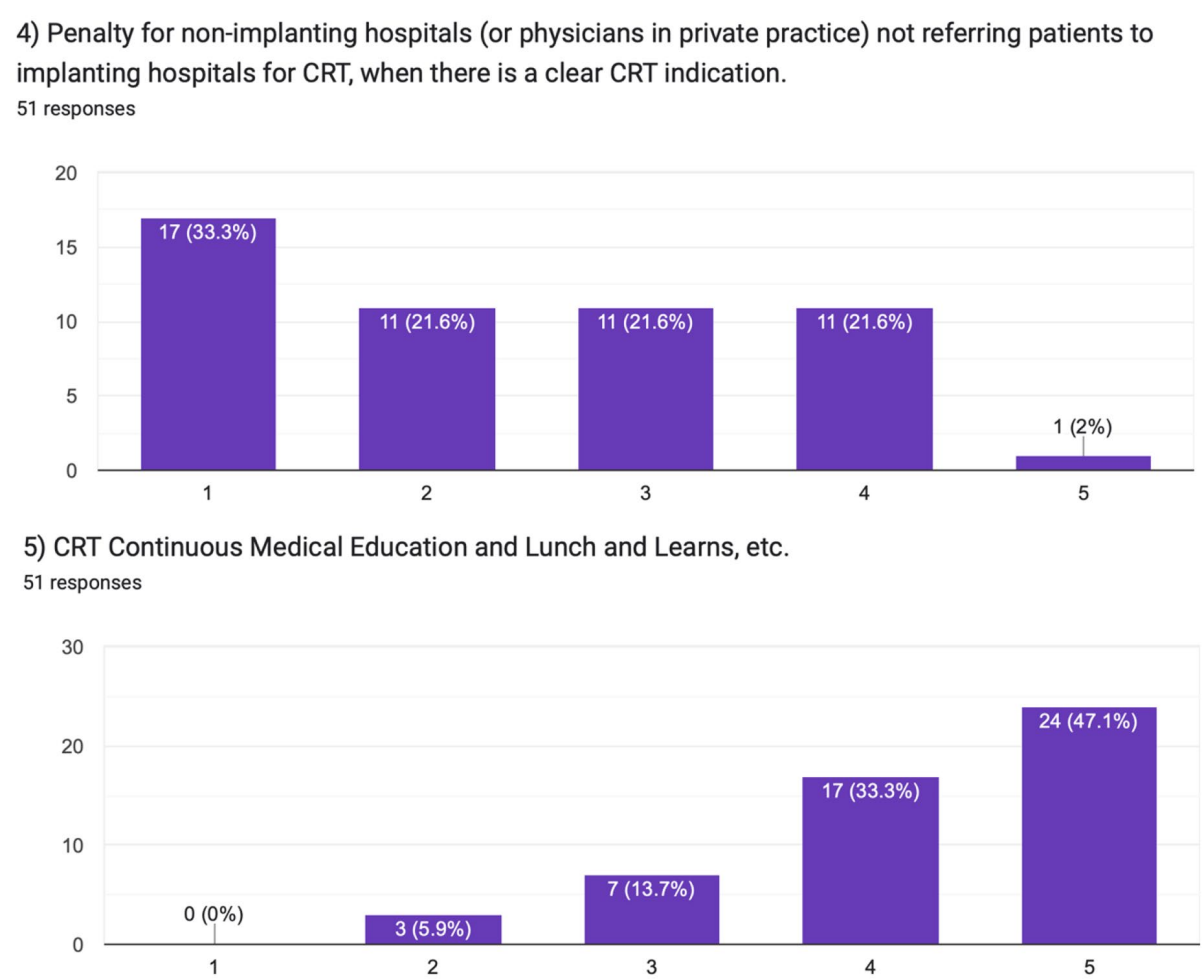
Pilot survey responses

The first question investigated the hypothetical impact of financial incentives for referring and completing CRT procedures (Fig. 2). A payment was proposed for referrals involving elderly and female patients, given their underrepresentation in CRT referrals. Participants regarded this monetary incentive as the least impactful. The median of the responses on the Likert scale was 2.0 [interquartile range, 1.0–3.0], where 1 indicates the lowest and 5 is the highest potential impact of the suggested intervention.

The fourth question addressed the consequences of penalties for non-implanting hospitals or private practitioners failing to refer CRT-eligible patients. Most responses indicated minimal impact. The median was 2.0 [1.0–3.0].

In contrast, the third and fifth questions explored the option of social incentives. The third question assessed the potential impact of publishing a mandatory, government-endorsed league table for CRT referrals and procedure execution. This proposition was perceived as highly effective. The median was 4.0 [3.0–4.0].

Similarly, the fifth question evaluated the effectiveness of continuous medical education and Lunch and Learn sessions in improving CRT referrals. Such an approach capitalises on the social dynamics of learning in a group of peers and the positive reinforcement that comes with continuous education, thereby fostering behavioural change. This intervention received a high rating - the median was 4.5 [4.0–5.0].

In the second question, the use of electronic patient records to prompt CRT referrals was rated. This measure employs a nudge and choice architecture. This subtly influences physicians’ decisions by making optimal choices (like referring eligible patients for CRT) more salient without removing any choices or altering economic incentives. The measure was rated as highly effective, with a median score of 4.0 [3.0–5.0].

When comparing the medians of the answers on the Likert scale given for the questions describing behavioural incentives (Questions 2, 3, and 5) and the ones describing traditional incentives, physicians were strongly in favour of the behavioural ones with 4.0 [3.0–5.0] versus 2.5 [1.5-3.0], *p* < 0.001. Despite the small sample size, response patterns appeared broadly similar across participating countries (Tables 1A and 1B).

**Table 1A:** Responses to behavioural incentive items

Country	*N*	Median	IQR	*P*
UK	14	3.0	3.00–5.00	0.240
Switzerland	12	3.5	2.25–4.00	
No country declared	10	4.0	3.75–4.00	
Poland	8	4.0	3.25–5.00	
Bulgaria	3	3.0	2.00 – NA*	
France	2	4.5	4.00 – NA*	
Ireland	2	4.0	4.00–4.00	
Total	51	4.0	3.00–5.00	

*NA indicates the 75th percentile could not be calculated due to limited data variation

**Table 1B:** Responses to traditional incentive items

Country	*N*	Median	IQR	*P*
UK	14	2.5	2.00–2.75	0.474
Switzerland	12	2.0	1.50–3.50	
No country declared	10	2.5	1.88–3.13	
Poland	8	1.75	1.00–2.50	
Bulgaria	3	2.5	1.00 – NA*	
France	2	2.5	2.00 – NA	
Ireland	2	3.25	3.00 – NA*	
Total	51	2.5	1.50–3.00	

*NA indicates the 75th percentile could not be calculated due to limited data variation

## Discussion

### Survey responses

We aim to elicit individuals’ attitudes towards behavioural and traditional incentives to increase CRT referrals among referring physicians. Compared to traditional incentives, our results reveal a significant preference among cardiologists for behavioural incentives over traditional monetary incentives. The significance of the finding lies in that it provides a foundation for alternative regulations and policies that call for further research into the underutilisation of CRT, specifically concerning behavioural interventions.

The questionnaire offered several other important insights for clinical practice design:

**1) Although financial incentives** are frequently regarded as a powerful motivator to change clinical practices, our results suggest that they are not necessarily well received or seen as effective in improving CRT uptake. Specifically, the response to the proposed financial incentive for referring elderly females was notably low (Question 1), with a median Likert score of 2.0 [IQR 1.0–3.0], indicating that participants generally perceived it as ineffective. This perception may be rooted in the existing **negative bias** toward referring elderly patients, especially elderly women, who could benefit from CRT [19]. Physicians’ negative bias is the *strong focus on the reported higher risk of interventional complications in general and a perceived limited benefit from complex interventions* in elderly patients [33]. As a result, offering a financial incentive without addressing these underlying biases may be ineffective and cause incentive backfire.

Financial **incentives can backfire** by appearing misaligned with physicians’ core concerns, as they fail to address the underlying reasons for their hesitation to refer this vulnerable population. Rather than encouraging referrals, monetary rewards could inadvertently deepen resistance, as the signal provided may seem incongruent with physicians’ intrinsic motivation and risk-averse approach to patient safety. Such incentives can *backfir*e when they are potentially perceived as external influences at odds with cardiologists’ won clinical judgment in high-risk cases [19, 25, 26, 34, 35]. Such a mismatch between financial rewards and physicians’ commitment to safeguarding patient welfare illustrates the limitations of monetary incentives in influencing referral behaviour for perceived high-risk groups.

In contrast, our findings suggest that addressing the negative bias directly, e.g., through strategies that reassure physicians of CRT’s safety and efficacy in elderly patients, may be a more effective approach than financial incentives alone. Examples include social incentives or choice architecture interventions could better align with physicians’ motivations and mitigate the influence of cognitive biases on referral decisions.

**2) Penalties** or negative financial incentives can fail to refer patients with a clear CRT indication, and consistently we find that they were rated as ineffective, with a median score of 2.0 [IQR 1.0–3.0] in response to **Question 4**, suggesting that traditional, blunt penalties do little to influence referral behaviour. Such penalties, applied broadly, fail to target specific cognitive biases that impact physicians’ decisions and might give rise to other undesirable behaviours to avoid the blame associated with them [19, 25, 26, 35].

In contrast, behavioural incentives using **loss aversion** and **commitment devices** offer a more nuanced alternative. If a physician does not meet a referral target, they return the incentive, which feels like a “loss” and effectively leverages loss aversion without external coercion. This approach encourages desired behaviour while preserving physicians’ autonomy, fostering a stronger commitment to referral targets. Traditional penalties assume that losing exerted the same effect as gaining something and hence do not take advantage of such bias [19],. By aligning with physicians’ professional values and intrinsic motivations, drawing on loss aversion can help promote CRT referrals more effectively, respecting the autonomy and judgement integral to clinical decision-making [19, 35, 36].

**3) Social incentives** were rated as highly effective in promoting CRT referrals, with the league table (Question 3) scoring a median of 4.0 [IQR 3.0–4.0] and continuous education sessions (Question 5) scoring 4.5 [IQR 4.0–5.0]. These results highlight the impact of **peer comparison** and **professional recognition** on referral behaviour. Indeed, leveraging **social standing** and professional **esteem** can steer physicians to align with peer norms of using CRT, and clinging to other peers’ practices can give rise to **social multiplier effects that generalise its use upon a certain level of CRT adoption**. Physicians may be motivated to increase referrals when referral rates are visible, especially when it enhances their professional image [19].

Similarly, continuous education sessions involving referring physicians both in outpatient practice and within hospitals capitalise on **peer interaction** and **network effects**, fostering a supportive environment to discuss the effectiveness of referral practices, showcasing successful CRT integration, and overcoming the inertia of following default practices. This aligns with norm persuasion, where respected peers’ examples can shift attitudes and behaviours. Such sessions encourage CRT referrals by fostering collective endorsement and addressing doubts about CRT efficacy, potentially reaching a **tipping point** for broader change [19, 28]. This may be particularly relevant in outpatient settings, where general practitioners often face greater administrative burden, clinical uncertainty, and fear of referral rejection. Strengthening relationships and communication between community providers and hospital-based specialists could further CRT referral pathways.

4) **Choice architecture** redesign by reminding physicians of the use of CRT when using electronic health records was highly rated for promoting CRT referrals, with **Question 2** yielding a median score of 4.0 [IQR 3.0–5.0]. This approach subtly influences physician decision-making by positioning CRT referral prompts as the **default option** within the electronic health record system, leveraging **status quo bias**—the tendency to follow readily available defaults. When optimal choices, like CRT referrals, are presented as the default path, physicians are more inclined to act on them [19]. Electronic health record prompts help position CRT referral as a benchmark action, becoming a salient reference point against judging their behaviours. In behavioural economics, deviations from these reference points are often perceived as losses, which enhances motivation to follow the prompt [19]. By framing CRT referrals as a default choice, choice architecture in electronic health records encourages alignment with best practices while preserving physicians’ autonomy in decision-making.

Electronic health records streamline the referral process, reducing friction and making CRT referrals the easier default option, reducing sludge or barriers in the system for their common adoption [19]. This aligns with the idea of recognising digital tools like electronic health records as effective aids for adherence to clinical guidelines, supporting physicians to make evidence-based choices with minimal effort. These findings suggest that a well-designed choice architecture in electronic health records can bridge the gap between guidelines and clinical practice in CRT referrals [19, 32, 37, 38].

### Behavioural incentive design strategies for CRT referrals

Based on the insights from the pilot survey and behavioural economics theories, a more comprehensive survey was developed to gather extensive data on referral behaviour and feedback on the proposed interventions (Supplemental Table 1). Below, we illustrate some behavioural concepts to understand the potential impact of varied behavioural interventions, allowing us to fine-tune our strategies further. Several strategies can be explored to enhance CRT referrals:

1. Maker use of commitment Devices and Loss Aversion: Encouraging some form of physician pre-commitment to refer a certain number of eligible patients for CRT in each timeframe (such as in the next six months), with an advance payment to all practitioners who commit; those who do not meet their commitment would need to return the payment, hence suffering a loss of an income loss that otherwise would be endowed, and therefore increasing the cost of inaction [19].
2. Prompting Reflection Reminders: Facilitating structured discussions or case reviews regarding CRT referral decisions during CRT Continuing Medical Education meetings and Lunch & Learns. This approach encourages physicians to critically evaluate referral decisions and identify potential biases or cognitive barriers associated with short-term memories [19].
3. Increasing Salience of CRT-related outcomes: Regularly share success stories or case studies of CRT outcomes (for instance, through a monthly newsletter) to increase its salience and enhance social effect. This strategy aims to inspire and motivate physicians to refer more patients for CRT by highlighting positive patient outcomes [19].
4. Narratives of CRT use: Utilising testimonials from peers who regularly refer for CRT, highlighting their reasoning and the positive patient outcomes they have seen. Peer testimonials can help create or consolidate social norms that encourage CRT referrals [19].
5. Meta-Nudges: Leveraging the influence of respected figures in the cardiology community (such as through public endorsements or social media campaigns) that use CRT. Physician endorsements from trusted leaders can influence referral behaviours of those individuals that role model them [19].
6. Patient and Public Involvement: Incorporating Patient and Public Involvement in developing and promoting CRT referral strategies can enhance patient engagement and improve retention in the CRT referral process. Such involvement strategies, including patient testimonials and public awareness campaigns, can streamline the CRT referral process by addressing patients’ informational and motivational needs and helping patients feel informed and valued in their care decisions. This approach not only supports patients’ active participation but also aligns with behavioural strategies aimed at fostering positive physician referral behaviour [39].
7. Default Options/Nudge: Make CRT referral the default option in health record systems for eligible patients (requiring physicians to opt-out if they do not wish to refer actively). Such nudge can promote CRT referrals by leveraging the status quo bias, taking advantage of routine and inertia [19].
8. Decision Aids/Simplicity: Develop decision aids (like flowcharts or algorithms) that outline CRT benefits and risks in a simple, concise manner. These aids can help physicians make informed and confident referral decisions [19].
9. Choice Architecture/Nudge: Streamlining the referral process (for example, by creating a one-click referral system within the electronic health record). Simplifying the referral process can remove barriers and increase CRT referrals [19].
10. Friction/Sludge: Increasing the complexity of opting out of CRT referral (for instance, requiring a written explanation or a review by a second physician when a physician chooses not to refer an eligible patient for CRT). This approach can discourage non-referral without imposing monetary penalties [19].

### Limitations

Based on voluntary participation and self-reporting, the pilot survey achieved a response rate of less than 20% among physicians. While physician surveys are valuable tools in health services and policy research, offering potential cost-effective insights into attitudes, knowledge, and practices, they often suffer from low response rates, which raises concerns about non-response bias and the generalisability of the findings.

While this study focused on behavioural drivers of CRT referral, we did not explicitly examine the influence of national policy frameworks or reimbursement strategies. The participating countries, Bulgaria, Poland, Ireland, the UK, Switzerland, and France, represent a diverse range of European healthcare systems and economic settings. All provide public reimbursement for CRT implantation, albeit with country-specific administrative frameworks. For instance, in Switzerland, a high-GDP country with social health insurance, the fee-for-service outpatient model may incentivise activity and facilitate referrals. In contrast, the UK, with a tax-funded system and comparatively lower GDP, applies global budgeting for outpatient specialist services and combines this with pay-for-performance mechanisms, potentially limiting CRT access [40]. Such structural factors may influence physician behaviour and contribute to variations in CRT uptake, as reflected in EHRA White Book data showing lower device utilisation in countries with lower GDP per capita [4, 13]. Nevertheless, the balanced representation of respondents across different healthcare funding models and GDP levels supports the broader applicability of our behavioural findings within the European context.

This study has further notable limitations concerning its small sample size, which may not represent the general perspective of the clinicians, a small number of questions, and the inability to determine the exact number of physicians approached. Furthermore, the environment can significantly impact the perception, e.g., private vs. public vs. academic institution, and physicians from different countries could have different perspectives. Additionally, the absence of follow-up questionnaires makes it difficult to assess changes in practice over time. A larger-scale survey is needed to more accurately evaluate perceptions of tailored incentives for increasing CRT uptake.

## Conclusion

The underuse of CRT in heart failure patients remains a critical healthcare challenge. Conventional strategies, such as financial incentives or guideline dissemination, have not improved CRT uptake. This paper presents both the foundational concepts of behavioural economics (such as choice architecture, cognitive biases, and social incentives) and evidence from a pilot survey suggesting that cardiologists’ attitudes towards these approaches are promising alternatives to traditional incentives. Specifically, strategies like peer comparison and decision prompts leverage behavioural insights to address knowledge resistance and support CRT referrals. By reshaping the decision-making environment, behavioural economics offers a nuanced framework for enhancing CRT uptake and improving outcomes in heart failure management.

## Electronic supplementary material

Below is the link to the electronic supplementary material.

Supplementary Material 1

## Data Availability

No datasets were generated or analysed during the current study.

## Abbreviations

CRT: Cardiac Resynchronization Therapy
LVEF: Left Ventricular Ejection Fraction
