# Supplementary material for: Behavioural insights in the underuse of cardiac resynchronisation therapy in heart failure: a pilot survey on incentive perceptions among referring cardiologists
Source: Health Econ Rev. 2025 Jul 18;15:62. doi: 10.1186/s13561-025-00657-0 (PMC12273364; doi:10.1186/s13561-025-00657-0)
Supplement: Supplementary file 1 — Supplementary Material 1 [file 13561_2025_657_MOESM1_ESM.docx]

# Supplemental Materials

## Table 1. Comprehensive Survey Questionnaire to be distributed Among ESC/EHRA, ESC/HFA members and CARDIOLOGYCASES.CO.UK subscribers

Section 1: Baseline and Demographics

1.1 What is your country of practice?

(Open-ended response)

1.2 How long have you been practising cardiology?

- Less than 5 years

- 5 to 10 years

- 10 to 15 years

- More than 15 years

1.3 Is your hospital/clinic equipped to perform Cardiac Resynchronisation Therapy (CRT)?

- Yes

- No

Section 2: Knowledge of CRT and Referral Practices

2.1 What proportion of your heart failure patients do you believe are candidates for CRT, according to the most recent European Society of Cardiology guidelines?

- Less than 10%

- 10% to 20%

- 20% to 30%

- More than 30%

2.2 What proportion of your heart failure patients do you refer for CRT?

- Less than 10%

- 10% to 20%

- 20% to 30%

- More than 30%

Section 3: Factors Influencing Referral Decisions

3.1 To what extent do you agree with the following statements regarding your decision to refer a patient for CRT (Scale: 1 = Strongly disagree, 5 = Strongly agree)

- I refer patients for CRT when they meet established clinical eligibility criteria.

- I consider the expected improvement in a patient's quality of life when deciding on a CRT referral.

- I weigh the potential risks of CRT complications when making referral decisions.

- I feel confident in my understanding of CRT indications and expected outcomes.

- The availability of institutional resources (staff, time, equipment) influences my decision to refer a patient for CRT.

Section 4: Knowledge Resistance and Behavioural Learning

4.1 Do you have all the necessary information to make informed decisions about CRT referrals? (Scale: 1 = Strongly disagree, 5 = Strongly agree)

4.2 To what extent do you agree that the following cognitive biases influence your CRT referral decisions? (Scale: 1 = Strongly disagree, 5 = Strongly agree)

- I tend to maintain my established referral practices. (Status quo bias*)

- I rely more on my clinical judgment over guideline recommendations when making CRT referral decisions. (Overconfidence bias*)

- I rely on recent or particularly memorable cases when deciding whether to refer a patient for CRT. (Availability bias*)

- My initial training and early experiences with CRT continue to influence my current referral decisions. (Anchoring bias*)

*The bias labels will not be shown to respondents but serve as a framework for analysing behavioural influences on CRT referral decisions.

Section 5: Traditional Incentives vs Behavioural Incentives

5.1 To what extent do you agree that the following factors could increase your likelihood of referring more patients for CRT?

(Scale: 1 = Strongly disagree, 5 = Strongly agree)

- I would be more likely to refer eligible patients for CRT if financial incentives or additional institutional resources (e.g., staff, equipment) were available.

- I would be more likely to refer eligible patients for CRT if structured behavioural strategies (e.g., reminders, peer comparisons, default referral settings) were implemented.

Section 6: Possible Strategies for Improving CRT Referrals

6.1 To what extent do you agree the following strategies could encourage more CRT referrals? (Scale: 1 = Strongly disagree, 5 = Strongly agree)

- Regular discussions of CRT referrals in team meetings or structured case reviews help improve referral patterns. (Counteracting Overconfidence & Status Quo Bias*)

- Sharing individual CRT responders' and real-world clinical CRT outcomes in professional meetings or publications increases confidence in CRT referrals. (Counteracting Anchoring, Negative Bias & Availability Bias*)

- Public endorsements of CRT referrals from opinion leaders (e.g., at conferences, in publications, or on social media) encourage referrals. (Meta-nudge, Leveraging Social Norms & Peer Influence*)

- Setting CRT referral as the default option in electronic health records increases referrals by requiring an active opt-out. (Leveraging Default Options/Nudge to make the CRT referral the easier choice*)

- Embedding a narrative or a slogan "CRT: Consider, Refer, Treat" into guidelines, education, and discussions to encourage proactive CRT referrals and reinforce timely uptake as standard practice. (Narrative*)

- Encouraging cardiologists to set a referral target for CRT-eligible patients within a given period increases referrals. (Leveraging Commitment Bias & Loss Aversion*)

- Streamlining the CRT referral process (e.g., a one-click referral system in electronic health records) increases referrals. (Reducing Sludge for referrals*)

- Documenting a reason in medical records for not referring eligible patients could improve adherence to CRT guidelines. (Introducing Friction for opting out, Nudges toward referrals by making opt-outs effortful*)

*The descriptions in brackets will not be shown to respondents but will serve as a framework for analysing behavioural influences on CRT referral decisions.
